# Ten simple rules for starting FAIR discussions in your community

**DOI:** 10.1371/journal.pcbi.1011668

**Published:** 2023-12-14

**Authors:** Frédérique Belliard, Angelica Maria Maineri, Esther Plomp, Andrés Felipe Ramos Padilla, Junzi Sun, Maryam Zare Jeddi

**Affiliations:** 1 TU Delft Library, Delft University of Technology, Delft, the Netherlands; 2 Erasmus University Rotterdam—Erasmus School of Social and Behavioral Sciences/ODISSEI, Rotterdam, the Netherlands; 3 Delft University of Technology, Faculty of Applied Sciences, Delft, the Netherlands; 4 National Institute for Public Health and the Environment (RIVM), Bilthoven, the Netherlands; 5 Faculty of Aerospace Engineering, Delft University of Technology, Delft, the Netherlands; Carnegie Mellon University, UNITED STATES

## Abstract

This work presents 10 rules that provide guidance and recommendations on how to start up discussions around the implementation of the FAIR (Findable, Accessible, Interoperable, Reusable) principles and creation of standardised ways of working. These recommendations will be particularly relevant if you are unsure where to start, who to involve, what the benefits and barriers of standardisation are, and if little work has been done in your discipline to standardise research workflows. When applied, these rules will support a more effective way of engaging the community with discussions on standardisation and practical implementation of the FAIR principles.

This is a *PLOS Computational Biology* Methods paper.

## Introduction

The FAIR data principles promote good data stewardship by leveraging the Findability, Accessibility, Interoperability, and Reusability (FAIR) of research data and software [1–4]. These principles aim to facilitate the discovery, access, integration, and reuse of research data and software by both humans and machines, with the ultimate goal of enhancing the transparency, reproducibility, interoperability, and impact of research. While the FAIR principles are not a single standard [5], they do emphasise the need for standardisation in the way research objects are described, stored, and shared. For this reason, the implementation of the FAIR principles often involves discussions over which practice, resource, or technology should be adopted as standard by a (research) community. By promoting consistent and well-defined data structures, controlled vocabularies, and metadata, the FAIR principles can help make research objects more easily comparable and reusable across different disciplinary and spatial contexts.

Despite the benefits of the FAIR principles and their widespread endorsement on behalf of research institutes, publishers, and funders, these principles have not been evenly adopted in all disciplines [6]. There is still a lack of data and code sharing (with estimates between 1% and 20% [7–11]—although there are higher sharing rates in, for example, genomic research [12]). Furthermore, not every discipline has access to metadata standards or discipline specific repositories. One of the main challenges to the wider implementation of the FAIR principles is linked to the social dynamics underlying standardisation processes. Standardisation is a complex process that involves the creation of agreed-upon rules across time and space ([13]; p. 71). This process is difficult to facilitate without sufficient leadership, resources, and time. Standardisation processes may also create frictions linked to imposing one solution on previously varied practices and to authority and governance issues (who decides on which standard to adopt?). These social dynamics are key to the successful implementation of the FAIR principles.

In this context, we shift our focus away from the specific research objects involved in standardisation processes and instead focus on the community aspect of standardisation. Specifically, we consider the strategies and approaches that can be employed to engage research communities in fruitful discussions about standardisation in the context of implementing the FAIR principles. In our view, the successful implementation of the FAIR principles relies on the buy-in and participation of the research community that will have to actually implement the principles.

**Fig 1:**
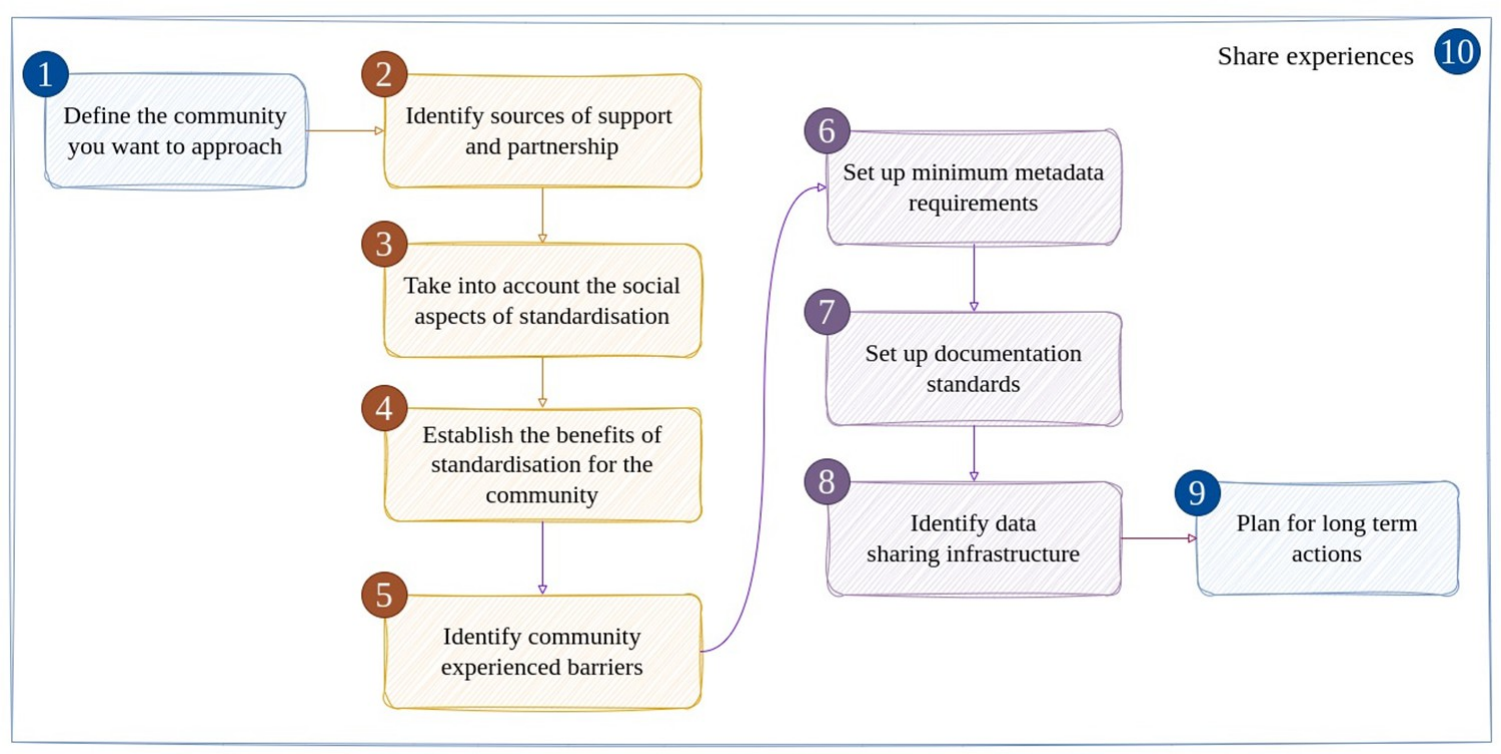
Overview of the 10 rules to starting FAIR discussions. It is important to define the community that needs to be involved (Rule 1) and gain partnerships and support (Rule 2). In these discussions, the social aspects of standardisation should be considered (Rule 3). It is therefore important to establish the benefits of standardisation processes (Rule 4) and to address the existing barriers (Rule 5). Keeping this in mind, it will become possible to set up minimum metadata requirements (Rule 6), documentation standards (Rule 7), and to identify the infrastructure that the community can make use of or should establish (Rule 8). In these efforts, the long-term sustainability should be considered (Rule 9). For each of these steps, it is important to share experiences (Rule 10).

To assist in the facilitation of standardisation discussions within individual research communities, we have developed the 10 rules as a reference point (see **Fig 1** for an overview). Input on these rules has been initially provided by experts (including researchers, data supporters, students, and service providers) at the Netherlands Open Science Festival on September 1, 2022 (see S1 Text for more details) and a call for contributions via FAIR connect [14]. It is important to note that not all research communities will be at the same stage of adoption of the FAIR principles, and some of these steps may be deemed unnecessary or irrelevant depending on the specific needs and circumstances of a given community. Our perspective will be biased towards the Dutch context as the Open Science Festival was hosted primarily for researchers in the Netherlands, and all authors are based at Dutch institutes. Nevertheless, we hope that these rules serve as a useful resource for researchers, Research Data Management (RDM) support staff, and research data infrastructure providers, looking to effectively promote the adoption of the FAIR principles within their own communities.

## Rules

### Rule 1: Define the community you want to approach

Discussions surrounding the adjustment of workflows to facilitate FAIR practices should occur within a research community, defined as a group of stakeholders (such as individual researchers, research support staff, and data infrastructure providers) that have a shared interest in streamlining their efforts to implement the FAIR principles. As explained by Timmermans and Epstein [13], standardisation is inherently a social process that requires the commitment and endorsement of multiple actors to be effective. The community aspects of FAIR implementation are embedded in the original FAIR principles [2] and made explicit in principle R1.3 (“(meta)data meet domain-relevant community standards”). For instance, in the framework of FAIR Implementation Profiles (FIPs, a methodology that has been introduced to document FAIR implementation choices), the community aspect is captured by the concept of FAIR Implementation Community [15,16]. How to adequately define and engage a community remains, however, an open challenge.

**Rule 1** recognises that identifying the appropriate research community is a crucial step in facilitating discussions on standardisation. The stakeholder who wishes to initiate a FAIR discussion may be already part of the community or not; regardless, it is important to provide a clear definition of what the community to approach is. Research communities can be based on various factors, such as the type of data being generated or used, a shared institutional affiliation, or a specific research project. A community can constitute a formal entity, or it can be an informal group, and it can exist for a determined time span, or be long-lasting [15]. It is important that a community self-identifies as such, as this can increase the level of commitment and engagement among members.

Research communities typically involve individuals in a variety of different roles, such as researchers, RDM support staff, lab technicians, and students. Once the community has been identified, the levels of understanding of FAIR implementation and FAIR standards should be gauged, using resources such as the FAIR-Aware tool developed by DANS [17] or the How to FAIR quiz from the Danish National Forum for Research Data Management [18]. Disparities in understanding among different stakeholders may present challenges to the standardisation process—though a diversity in perspectives can be beneficial, as elaborated in **Rule 3.**

Depending on the features of the community, there may be different ways to get in touch with the community members: In the case of an informal community, for instance, it may be necessary to proceed via “snowballing,” with one identified member suggesting other ones and so on. In the case of formalised communities, instead, there may be people with specific roles (such as community managers) who already have open communication channels with the community. In both cases, reaching out to RDM experts, research infrastructures, or scientific associations may be beneficial (see **Rule 2**), as they may be aware of existing or similar initiatives, or be able to suggest people to contact.

### Rule 2: Identify sources of support and partnership

Attempting data standardisation is a complex process that should not be done alone. Support and partnerships are most likely to be found from the RDM support team at your institute (usually based at the library), the scientific association of your discipline (see **Table 1** for some examples), or other enthusiastic individuals already involved more closely with the adoption of the FAIR principles. Failing to engage with these stakeholders may result in a lack of awareness and recognition of the need for promoting the FAIR principles in your community within your institute or association. We recommend prioritising seeking out this type of support or partnerships, as it could prove to be beneficial in the long run, even if funding or resources may not be immediately available.

**Table 1:** Examples of organisations that can help finding RDM experts grouped by spatial focus (primarily in Europe) and domain specificity. The examples are meant to give an indication, not an exhaustive overview. This overview is available at https://github.com/AngelicaMaineri/awesome-RDM-support/blob/main/README.md under a CC0 licence to allow reuse and extension.

Focus	Domain specificity	Examples
Institutional level		(Digital) competence centres - university libraries - university research support team - institutional/faculty/department data steward Open Science Communities (see International Network of OSC)
National level	Domain-specific	Research Infrastructures, scientific associations: - ELIXIR’s national nodes (life sciences) - Italy’s Foster Open Science in Social Science Research (FOSSR) - the Dutch Open Data Infrastructure for the Social Science and Economic Innovations (ODISSEI)
	Domain-agnostic	Data archives, scientific associations: - National data archives (see for examples the list of contributors to the CESSDA data management guide or the Australian Data Archive - ADA) - Research Data Alliance (RDA) National groups - Research Data Access and Preservation (RDAP) RDM national initiatives: - the Netherland’s LCRDM (see LCRDM’s pool of experts) - the Netherland’s Data Stewardship Interest Group (DSIG) - Italy’s Italian Computing and Data Infrastructure (ICDI) Competence Center - UK’s Data Curation Center (DCC) - Denmark’s National Forum for Research Data Management
International level	Domain-specific	Research Infrastructures: - Consortium of European Social Science Data Archives (CESSDA) - ELIXIR, a European infrastructure for the life sciences
	Domain-agnostic	Scientific associations and interest groups: - RDA’s working and interest groups - RDA/EOSC Future ambassadors - GOFAIR Implementation Networks

If you are a researcher, start by checking if an RDM team is available at your institution. The RDM team will be able to point to existing resources, tools, and information that can save time. The RDM team can also provide support in raising awareness, as they should already be involved with promoting the adoption of FAIR principles within the research community. This RDM team will likely have experience with providing workshops, training programmes, setting up policies and recommendations, and hosting events. The RDM team may have already set up materials or resources that can be tailored to specific needs. Vice versa, if you consider yourself as an RDM specialist or an infrastructure provider, make sure to seek partnership with researchers in the community, as they can contribute their domain-specific knowledge as well as their perspectives of potential data (re)users. Regardless of your role, the scientific association or individual researchers already involved with the FAIR principles may have already set up data standardisation processes that you can join, will provide connections to your community (see **Rule 1**), and can provide further support.

Ideally, there is funding involved in standardisation efforts. Both RDM support and scientific associations may also have access to funding, or they may be able to connect you to other funding opportunities. Funding agencies in the Netherlands allow for funding data management activities in their projects (NWO and ZonMW). Open Science Funds may be available through Open Science Communities or at a (inter)national level (see NWO or FAIR impact).

Once you have defined your community and found further support, the next step is to consider the social aspects of standardisation processes (**Rule 3**).

### Rule 3: Take into account the social aspects of standardisation

As discussed in **Rule 1**, adopting research workflows is a social process that requires the participation and commitment of multiple actors within a research community [13]. Standardisation efforts can stop or become obsolete when a community loses interest [19]. Therefore, **Rule 3** highlights the importance of considering the social aspects that may influence the consensus-building process when facilitating discussions on the adoption of FAIR practices and offers practical examples. The rule is quite broad in its formulation because the way in which these social dynamics manifest themselves may vary depending on the community and the stakeholders involved. We aim to illustrate some potential situations and actionable recommendations.

First of all, the creation and adoption of standards may involve trust and authority issues. For example, the introduction of new standards may generate opposition and resistance if community members do not perceive them as legitimate or do not trust the authority of those proposing the standards. To mitigate the risk of frictions, involving the community is key. In particular, highlighting best practices via real use cases from the community can be effective in showing the value of standardised FAIR practices and promoting the use of existing standards. Generating trust in the process can be facilitated by having regular meetings, including occasional in-person meetings if possible, and by planning clear feedback processes so that concerns from the community can be addressed.

When there are no existing best practices yet within your own community, examples and recommendations can be adopted from other communities who are further along in adopting the FAIR principles (see **Rule 2**: RDM experts can help reaching out to more mature communities; but also see **Rule 10**: Sharing experiences is pivotal!). Building on existing practices may save time and resources that can be used more efficiently in the standardisation process.

Additionally, standardisation can sometimes result in a reduction of heterogeneity within a community, as it involves the creation of agreed-upon rules that may limit the range of previously enacted practices and even supersede previously adopted, perhaps outdated, standards. Therefore, it is important to carefully consider the potential impacts of standardisation on the variety of research practices within a community. The community-based approach to the FAIR principles, alongside the FIPs, which support the documentation of FAIR implementation choices [16], makes it possible to create multiple standards, as long as cross-standard interoperability is kept into account. For instance, the CEDAR Metadata Tools allow the creation of community-specific metadata templates while reusing existing ontologies and value sets, therefore enabling diverse solutions within a shared framework (see **Rule 6** for more recommendations on creating metadata models). There are also solutions to make existing standards more easily findable and reusable. One such solution is FAIRsharing, which serves as a repository of FAIR-enabling standards and other FAIR resources. This platform was created to address the issue of excessive fragmentation in the developments of standards [20].

Standardisation often requires researchers to invest time and resources into changing their existing workflows, which can be a challenging task. The disruption of existing practices has also been reported to be an important barrier to adoption of standards in the industry context (see [21] and **Rule 5**). Therefore, it is important to clearly communicate the benefits and incentives of adopting the FAIR principles to the research community. By clearly communicating the value of the FAIR principles and engaging in meaningful dialogue with the research community, it is possible to facilitate a more effective and efficient standardisation process (see also **Rule 4**).

### Rule 4: Establish the benefits of standardisation for the community

It can be helpful to establish benefits of standardisation in order to convince others to get involved and motivate them to change their workflows. Some examples of benefits are listed below and may not always be directly applicable to your community.

Personal benefits by being involved in this process include:

- Direct impact on the eventual results, ensuring that the standardisation processes are applicable to your research.
- Extension of professional network and positive effects on your professional reputation.
- Long-term standardisation can be more cost efficient by increasing data reuse, preventing duplication of research/trials, improving quality of the data (reduction of data errors and increase of reproducibility), and facilitating integration of datasets.

FAIR standardisation processes also reflect positively on institutions (see **Rule 2** on where to find help in your institute). Benefits for institutions include:

- Streamlining data management processes and being cost efficient.
- Increasing the reputation and trust in research findings from the related research groups [22].
- Rewarding and recognising data as a valuable research output in academia.
- Facilitating collaboration and innovation [22].
- Increasing the value of existing data by facilitating reuse and, thereby, increasing the return on the initial investment on data collection.

After the benefits have been established, the next step is to identify the barriers (**Rule 5**).

### Rule 5: Identify community-experienced barriers

It is important to recognise that the barriers and challenges to implementing the FAIR principles and standardising data management practices may vary widely across different research domains and communities. This may involve performing a gap analysis to identify areas where additional support or resources are needed for the community (identified in **Rule 1**), or identifying case studies of successful FAIR implementation in similar disciplines that can serve as best practice for the community (see also **Rule 3**). Below are some of the barriers that we have encountered, either in the literature or in our own professional experience:

- **Data requirements**: The types of data more commonly used by a community as well as their size and heterogenous nature may pose challenges in terms of storage, management, and accessibility [23–25].
- **Ethical and legal barriers**: The handling and sharing of sensitive political or personal data may be subject to strict regulations (such as the General Data Protection Regulation (GDPR)) and require additional considerations to ensure compliance [9,23–29]. Data sharing can also result in economic damage when disease data are shared and impact tourism and trade [29].
- **Different research environments**: When there are critical resource shortages (such as the absence of research networks and lack of infrastructural support), there may be more immediate concerns that should be addressed [30].
- **Intellectual property and licensing**: Intellectual property (IP) issues (such as data transfer and processing agreements) may arise when data are shared or reused [9,24,25], particularly when multiple stakeholders are involved. Not everyone may have access to proprietary software used in data analyses.
- **Lack of incentives**: Some researchers may not see the value in making their data FAIR or may not perceive a need to share their data with others (see **Rule 3**) [23,24,26–29,31–33].
- **Cultural barriers**: for example, considering data sharing to hamper future publications if there is no reciprocity in the form of appropriate credit for data sharing [25,26,29,31], or a lack of trust in data being correctly interpreted and used [23,24,27–29,32].
- **Lack of institutional data policy, support, and training** [34].
- **Lack of infrastructure** (see **Rule 8**) to share data [23,25,29,31,32].
- **Lack of compliance monitoring** by institutes, funders, or journals with policies regarding the FAIR principles, decreasing the need for researchers to comply with these policies [9,23,32,35].
- **Limited awareness** about best practices, FAIR principles, and standards [28].
- **Emphasis on novel research** may result in data generation rather than reuse, integration, and maintenance [33].
- **Possible criticism** and fear that results will be invalidated [23,29].
- **Limited time and/or lack of resources** [9,23,25–29,32,34].

To identify and address these barriers, you should discuss them with your community (**Rules 1** and **3**).

Pending on the identified barriers, there will be different solutions to address them. Some of these barriers (limited awareness, lack of expertise and best practices) can be addressed by data standardisation and defining more explicitly what information is most valuable in the data management workflows. In **Rule 6,** this is addressed by going deeper into how information requirements can be established.

### Rule 6: Set up minimum metadata requirements

Metadata is information about the data that provides context and allows for proper interpretation and reuse. A metadata standard is a structured form of documenting and describing this information. Several metadata standards are already in use, such as Dublin Core and DataCite ([36]; see the Digital Curation Center for an overview of metadata standards, or use FAIRsharing to browse metadata standards). Dublin Core consists of 15 general elements that make this standard easy to use across disciplines. Nevertheless, most disciplines and research communities may require more detailed metadata than those provided by Dublin Core in order to manage and document their research data effectively. This will eventually result in data shared in research repositories that are better aligned with the FAIR principles. It is therefore helpful to look for discipline-specific metadata standards or guidelines (see FAIRsharing.org [20] or the Metadata Standards Catalog). When there are no metadata standards or minimum metadata requirements available for your community, a more advanced step is to start creating these minimal metadata requirements. This can be a complex task, particularly when your community spans over different fields that use distinctive terminology to describe data [37].

To start with setting up minimum metadata requirements, it is important to first establish who needs to be involved, as community engagement will be crucial [37] (**Rule 1**). You may also need to consider who is most suitable to lead this process, as this will require some degree of authority, expertise, and trust within your community (see **Rule 3**). Other communities have already been successful in developing minimum metadata requirements, such as the Earth Sciences [38,39], Bionano Sciences [40], Biomedical Sciences [41], and -omics Sciences [42–45]. The lessons learned from these communities can be taken into account, although their approaches may not always fit with your research community that may have different requirements and challenges (**Rule 5**).

Minimum metadata requirements can be about data/sample preprocessing, experimental analysis, quality control, preregistration—any aspect related to the research process. The metadata requirements should provide guidelines for essential information requirements while at the same time be flexible to meet each researcher’s objectives [41,46]. There is a need to “strike the right balance between minimising the barriers to data submission and maximising opportunities for data reuse” [41].

After you identified the relevant stakeholders (**Rules 1** and **2**), you can follow the recommendations below to start setting up minimum metadata requirements, or a Minimum Information Standard, in a research community:

- Review existing practices such as metadata standards, guidelines, and use cases [38,39,42,43,46].
- If there are no existing efforts, you can start with a call for guidelines [44], set up a working group/project team [39,45], or a network [47]. You can get a team together by organising a workshop or conference session [41,42,48,49]. Ideally, funding is available for standardisation efforts (for example, NIH funding [19]) or should be applied for [47,48] (see also **Rule 1**).
- Adhering to the minimum metadata requirements should be as effortless as possible to enable widespread adoption [46,48] (see also **Rules 3** and **4**).
- Minimum metadata requirements are the first step towards standardisation. Additional developments will be needed for standardisation [42,46], involving the research community at each step.
- To establish community consensus, the research community should be asked for input and feedback, through community discussions, workshops, and surveys [38,41–44,48]. Only through active community involvement will a functional solution be achieved [45] (see also **Rule 3**).
- To ensure practical and effortless implementation of the standards by journal editors, reviewers, and data repositories, it is important to gather their feedback [39,41,45,49].
- Once progression has been made, it is important to communicate this to the research community, via public documentation, reports, or publications (see also **Rule 10** on sharing experiences).
- To support community uptake, it can be helpful to provide training, support, or to have champions involved that can promote the standards [38]. The benefits (**Rule 4**) of adjusting existing workflows should be clear.

A great way to get started is to review the work by ESS-DIVE in establishing a community-centric metadata reporting format [38]. Crystal-Ornelas and colleagues [38] share guidelines (Box 1) and details about their process. A next step could be to develop an ontology (see Courtot and colleagues [50]’s 10 simple rules on this topic).

### Rule 7: Set up documentation standards

In addition to the minimum metadata requirements described earlier (**Rule 6**), documentation is the next step that supports reuse of research outputs. The type of documentation needed depends on the purpose, expertise, and context of your community. If the primary purpose of the documentation is to be published in a research repository (see **Rule 8**) or to comply with the funders’ policies, the documentation must, at a minimum, be designed to achieve that purpose. Two general levels of documentation can be considered when documenting research data: project level and data level (also referred to as study-level and object-level documentation, respectively). The project-level documentation provides context for the collection, methodology, structure, and validation of data, while the data-level documentation consists of the variable names, descriptions, classifications, file formats, and software details. In other words, project-level documentation is about what is around data, and data-level documentation is about data itself [51].

Examples of project-level documentation are Data Management Plans (DMPs), Software Management Plans (SMPs), and the use of Preregistration and Registered Reports. The first two provide project-level documentation by describing the context of data and software; in DMPs, describing how data were collected and the methods used to validate it [52]; in SMPs, describing how software works, the purpose, the outputs, and its (continuous) development. Research communities may select standard templates for DMPs, taking into account the requirements of their organisation or funder (see for examples the list of public templates on DMPTool), while for SMPs, such standards are under development [53]. Preregistration, on the other hand, involves the public disclosure of research plans before data collection, analysis, and reporting are completed, with the goal of increasing transparency in the knowledge creation process from its inception to the results [54,55]. In an effort to standardise the information requested for preregistering a study and ideally simplify the process for researchers, templates have been developed (see the list of templates on the Open Science Framework website).

At the data level, once the minimum metadata requirements are established (**Rule 6**), it will be easier to describe variables or file formats that the community will use and then expand to documentation guidelines. Documentation can be used to describe how to organise data, such as spreadsheets [56], workflows (see the Data Curation Network Primers), and to provide information on standards for dates and times (such as ISO 8601 or RFC3339). In addition, it may be possible to use documentation from other communities. In particular, code programming communities use standard style guides that are also widely used within research communities (such as PEP 8 for Python, Tidyverse for R; see also guidance by the Code Refinery [57]).

A recommended solution for documentation could be codebooks, which describe the variables with their units, summarising choices made during the research process, and outlining the experimental study design [58]. Ideally, codebooks should be in a structured/standard format (for example, Data Documentation Initiative Codebook). Recently, tools have been developed that can automatically generate standardised metadata, reducing the (time) barriers to writing comprehensive codebooks (codebook R Package [59]).

Ultimately, the choice of documentation standardisation should facilitate communication and collaboration between researchers and those who reuse their data. **Rule 8** outlines the process of choosing the infrastructure to share the data and the accompanying documentation.

### Rule 8: Identify infrastructure to share data

In order to get a clear idea of the infrastructure that can be used to share data, first, it is important to follow the requirements and guidelines of your institution, funders, and/or collaborators. Generally, data repositories are considered to be the ideal infrastructure to share data in a reliable manner [60]. Generic repositories, such as Zenodo, OSF, or Figshare, are widely used for preserving and sharing research data (see **Table 2** for some examples). Your institution may also already have a repository that you can promote within your community.

**Table 2:** A list of common repositories outlined in more detail in [61].

Repository	Dataset limit	Host	Comments	Certification
Zenodo	50 GB	CERN (CH)	General-purpose repository	No
Figshare	20 GB 5 TB (Figshare+)	Digital Science (UK)	General-purpose repository	ISO27001
Harvard Dataverse	1,000 GB	Harvard University (US)	General-purpose repository	No
Dryad	300 GB	Dryad Digital Repository, Inc. (US)	Popular for research data underlying scientific and medical publications	CoreTrustSeal
4TU.ResearchData	100 GB	4TU.Centre for Research Data (NL)	Popular for technical and scientific research data	CoreTrustSeal
Open Science Framework	50 GB (public) 5 GB (private)	Center for Open Science (US)	Information and data sharing platform	No
Mendeley Data	10 GB	Elsevier (NL)	General-purpose repository	No

Generic and institutional repositories are generally not designed with the needs of a specific community in mind, which is where discipline-specific infrastructure may play an important role. Discipline-specific infrastructure is especially beneficial if standard data formats are used and enforced, ideally via user-friendly interfaces and with training provided where needed [62]. There are many domain-specific repositories (see NIH list of domain-specific repositories). It is therefore important to determine which type of data repository will better serve the needs of your community. Communicating a preferred infrastructure to share data may result in data that are more findable (as data are shared using the same infrastructure) and may reduce the cognitive load for individual researchers within your community as they do not have to look for suitable data repositories themselves.

Before sharing data via a data repository, or promoting your repository to the community, you will need to verify that the repository follows the minimum requirements to be considered useful and adheres to the FAIR principles. Think that a repository needs to:

1. Have a clear policy on how data will be managed, as well as a privacy policy and terms of use.
2. Provide sufficient data storage size for the dataset.
3. The geographic location where the data are saved (for restricted access dataset that contains personal data).
4. Assign a persistent identifier (such as digital object identifier (DOI)) to be able to cite the data.
5. Allow you to include a licence to your data (such as a Creative Commons licence).
6. Make sure data are available/accessible and discoverable. Repositories can enhance their discoverability by being included in databases such as re3data (https://www.re3data.org [63], FAIRsharing (https://fairsharing.org [20]), and the EOSC portal (https://eosc-portal.eu).
7. Allow revisions to be made to the dataset in the future.

In some cases, institutional or generic repositories do not fulfil the requirements of your community (see **Rule 5**), and there may not be a discipline-specific data repository available. Setting up a specific repository may be a good option when there are sufficient resources and plans for the long-term sustainability of the infrastructure (see **Rule 9**). The main advantage of arranging your own repository infrastructure is that you have greater control over how data are documented and presented to the public and/or researchers. Specific data repository infrastructure may also improve the data quality of the datasets [64]. However, creating and using this infrastructure leads to additional costs (especially when dealing with large quantities of data). In addition, clear documentation and training materials (see **Rule 9**) are required to engage researchers to use the repository.

### Rule 9: Plan for the long-term actions

As mentioned in **Rule 8**, discipline-specific infrastructures require resources and should be sustainable for the long term. When these infrastructures are set up by a small group, maintenance and sustainability is challenging as many researchers move across institutes and countries. While sustainability can be achieved by charging for repository services, it is also important to consider that not all researchers have access to these resources. Researchers are generally working on projects that eventually run out of funding, especially at the stage of data sharing. It is therefore important to consider who pays for long-term data sharing and maintenance. Maintenance plans and governance of infrastructure and standards should be transparently communicated (see also **Rule 3**).

To plan for the long term and to establish robust policies for the repository, repositories could aim for certification (via CoreTrustSeal, ISO 16363, or Nestor), although this is a resource-intensive process. Resources are also needed for the maintenance of any created metadata standards (**Rule 6**). Standardisation is a continuous process and will require evaluation on their practical applicability (for example, metadata standards may become obsolete or deprecated when they are no longer applicable [19]). Standardisation is also a continuous learning process. Researchers may not be familiar with the standardisation efforts and will need a place to start, support, or training resources. It can also be important to monitor whether standardisations are followed appropriately—some form of manual curation may always be needed to avoid errors or incomplete entries. All of these processes take up resources in the long term.

To facilitate long-term sustainability, it is better to use open formats and infrastructure built using open source software. This prevents lock-in to certain services and allows community members to continuously contribute. It is also important to consider how the infrastructure will scale when future use is increasing and user input may become more heterogeneous [65].

Individual researchers can improve the longevity of research data by starting to make use of data repositories and make their data available in open formats, following the repository guidelines. When researchers are already using data repositories, they can promote the use of data repositories to their colleagues and share any available training materials—especially if they are in leading roles or responsible for the training of other researchers. Individual researchers can also financially support data repositories by including a budget for data curation in their research proposals or by requesting funding from their institutions for data curation. Individual researchers, or research data support staff, can also provide other types of resources to data repositories by performing data curation tasks, data peer review, or taking on communication tasks.

To foster a culture of data standardisation and sharing, it is needed to recognise the efforts of researchers who adopt minimum metadata requirements (**Rule 4**). Ideally, this happens at the institutional level. Research communities can also recognise practices during annual meetings and conferences or by awarding prizes (for example, the Open Science Community Amsterdam Awards that took place on January 26, OSCA 2023).

### Rule 10: Share experiences

Sharing experiences about the standardisation process facilitates learning from existing efforts and identifying best practices that can facilitate the standardisation journey. By reaching out to local RDM support or other community stakeholders (see **Rules 1** and **2**), you have hopefully benefitted of the experiences of others as well. It is therefore important to share experiences gained from each of the rules listed here and as illustrated in **Fig 1**. Experiences and insights can be shared via case studies, best practices, and lessons learned from standardisation efforts. Venues to share these experiences may include journals (such as PLOS), preprint servers, publishing forums (such as FAIR connect, where an earlier version of this article was shared [14]), data repositories (such as Zenodo), blogs (for example, [66]), social media, or conferences and meetings.

## Conclusions

Adopting the FAIR principles and adjusting research workflows is a complex and time-consuming process. The recommendations that we share emphasise the need to identify the community that needs to be involved (**Rule 1**) and find support as well as the relevant stakeholders that need to be involved (**Rule 2**). As adjusting existing workflows is primarily a social issue (**Rule 3**), it is important to identify the benefits (**Rule 4**) and to address the existing barriers (**Rule 5**). Keeping this in mind, it will become possible to set up minimum metadata requirements (**Rule 6**), documentation standards (**Rule 7**), and identify the infrastructure that the community can make use of or should establish (**Rule 8**). It is important for infrastructure, and also for metadata standards, to consider the long-term sustainability of the efforts (**Rule 9**). Crucial to each of these steps is the sharing of the lessons learned and the materials created so that others do not have to start from scratch (**Rule 10**). By following these recommendations, you should be able to more successfully engage your community in discussions that will result in successful implementations of the FAIR principles.

## Supporting information

S1 Text Process to getting to “Ten simple rules for starting FAIR discussions in your community.” (DOCX)
