# Supplementary material for: Ten simple rules for starting FAIR discussions in your community
Source: PLoS Comput Biol. 2023 Dec 14;19(12):e1011668. doi: 10.1371/journal.pcbi.1011668 (PMC10721007; doi:10.1371/journal.pcbi.1011668)
Supplement: S1 Text — (DOCX) [file pcbi.1011668.s001.docx]

# Supplemental Materials

## S1 Text. Process to getting to ‘*Ten simple rules for starting FAIR discussions in your community*’

Starting FAIR discussions in your community was originally conceived as a session for the Open Science Festival 2022 by Esther Plomp. After internal discussions at TU Delft between Esther and Frédérique, and a call for contributions on Twitter, the session was submitted by 13 May 2022 (see **Table S1** for the proposal text).

**Table S1**: Session proposal submitted to the Open Science Festival 2022.

| **Information fields of the submission form** | **Answers by the contributors of the session proposal** |
| --- | --- |
| Names of organiser(s): | Esther Plomp, Delft University of Technology - Faculty of Applied Sciences  Frédérique Belliard, Delft University of Technology - Library  Junzi Sun, Delft University of Technology - Faculty of Aerospace Engineering  Esther Maassen, Tilburg University - Faculty of Social and Behavioral Sciences  Angelica Maineri, Erasmus University Rotterdam - Erasmus School of Social and Behavioural Sciences |
| Names of moderators / speakers if applicable; | Moderators: All organisers, pending on how many participants will join the session to form smaller groups. |
| please indicate if you want to organize a workshop or session: | Session (45 min) |
| Short description that summarizes the workshop / session, its rationale and relevance for the participants of the National Open Science Festival; | For data to align with the FAIR principles it has to follow domain-relevant standards (principle R1.3). Nevertheless, not all disciplines have metadata standards or standardised workflows, and the knowledge thereof varies considerably across different stakeholders. This session aims to exchange experiences on increasing standardisation in one’s research field. These experiences and recommendations will be translated to a short guide/checklist for anyone that wants to start standardisation discussions within their research community. Questions/steps to provide information on could be ‘how do we engage all the stakeholders?’ and ‘What would be the best way to structure/start these types of discussions?’ or ‘Is it possible to obtain funding for this?’  This aligns well with the festival’s proposed topics of:  - Putting FAIR and open data into practice  - Setting up and maintaining open communities  - Working-sprints with peers on a specific open topic |
| Format and structure; | This session will be hosted as a discussion session in which participants will be split into smaller groups to brainstorm and gather input. These inputs will be gathered collectively. Interest will be gathered during the session for any follow up steps at the end.  xx:00: Introduction to the topic and determining level of experience with the topic  xx:05: Grouping of individuals with different experiences into smaller subgroups so that they can discuss  xx:25: Short report out of the progress  xx:30: Gathering initial checklists/steps and repeated points  xx:40: Next steps  xx:45: End of session |
| Target audience; | - Researchers that would like to start standardisation discussions in their own discipline.  - Research professionals that would like to facilitate standardisation discussions.  - Representatives of professional societies, data repositories, or funding agencies, and editors |
| Preferred outcomes for participants of the workshop / session; | Participants will build up their network and get to know individuals with a similar interest in data standardisation  Start of a short guide/checklist which will be shared on a data repository once it is finalised (with contributors credited for their work) |
| Requirements for the workshop / session (screens, set up of tables, flipcharts, etc.) | Flipcharts  Tables set up in small groups for discussions |

Due to a role change, Esther Maassen could unfortunately no longer dedicate time to the session.

The session was accepted and advertised on the Open Science Festival 2022 website as following:

**1 September 13:45 – 14:30**

**Title: Starting FAIR discussions: increasing standardisation in your research community**

**Abstract:** This session aims to exchange experiences on increasing standardisation in one’s research field. These experiences and recommendations will be translated to a short guide/checklist for anyone that wants to start standardisation discussions within their research community. Questions/steps to provide information on could be ‘how do we engage all the stakeholders?’ and ‘What would be the best way to structure/start these types of discussions?’ or ‘Is it possible to obtain funding for this?’

During the session preparations the schedule for the workshop was adjusted as following:

- xx:00: Introduction to the topic and determining level of experience with the topic
- xx:05: Subgroups (data collection, standardisation in publishing, community engagement, data sharing)
- xx:20: Switch from problem reporting to steps to take/solutions
- xx:35: Post stickies
- xx:40: Next steps (invite them to session on 8th of September)
- xx:45: End of session

The session was led by Frédérique Belliard, and facilitated by Junzi Sun and Angelica Maineri. After a brief introduction on the aims of the session (to co-create a checklist for anyone who wants to start FAIR standardisation discussions within their research community) the participants at the session split into five round tables. Four of these tables had predefined topics identified by the session organisers: data collection; standardisation in publishing; community engagement; and data sharing. The fifth table was suggested by the audience; Reusability. Recommendations developed at the tables were then shared via post its on a board. Participants were asked whether they were interested in further contributing to the checklist/10 rules via an online meeting taking place a week after the festival on the 8th of September.


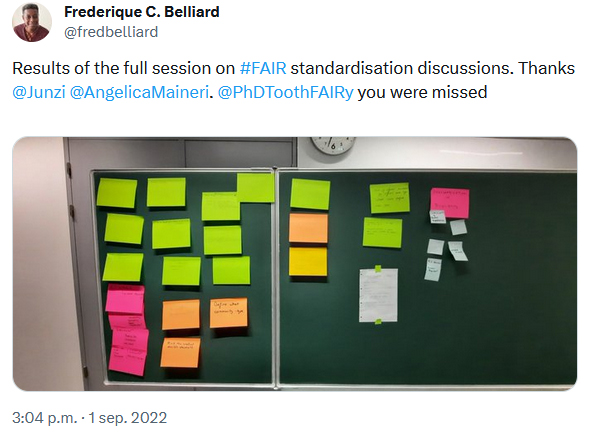


[Tweet by Frederique Belliard.](https://twitter.com/fredbelliard/status/1565324621116805125)

After the meeting on the 8th of September the group was extended with Andrés Felipe Ramos Padilla and Maryam Zare Jeddi. We discussed the session input (see the overview of the contributed stickies below). We started meeting regularly to work on the article via optional online co-working calls and discussion sessions until submitting the article in April 2023.

## Summary of the stickies from the OSF session

**Community engagement**

- Define what community type
  - *In the community engagement table, some elements were mentioned that “make” a community: self-identification; sharing data types; institution*
- Community consensus on the path of data sharing
- Community momentum on creating domain specific metadata and publishing standards
  - *In the community engagement table, a point was raised that it can be effective to learn from communities that are further along in the FAIR standardisation process (Use cases?)*
- Policies for the institutes/projects about data sharing. For example, what to do when the owner/researcher goes away.

Data ownership could be transparent so it is not lost

standard in data usage policies

how to automate usage policies on infrastructure

- how to control policy violations

Storage platform - unified? Or everyone keeps their data?

What are the terms of use - licenses should be clear

Data sharing: standardisation, also documentation

Problems

- data sustainability: what happens at the end of the funding/project
- ‘My data isn’t good enough (yet) for sharing’
- Terms of use unclear
- metadata: community-specific requirements

Data sharing

- long-term planning: who is responsible (ownership), who will pay for keeping the data available?
- Where to share data/publish? Generic, discipline specific?
- Discipline specific documentation:
  - what information to provide to future (re)users
  - how to name your variables?
  - how to organize your files?
  - how can others (re)use data what can they do with it?
  - how were the data created

Follow the FAIR principles -> make the data as FAIR as possible

create a checklist for starting a conversation

- metadata catalog
- dmp
- standardisation vs harmonisation?
- ontologies
- it should not constrain science
- clear definitions of words. know your synonyms
- emphasize the benefits
- if there is no standard, document all your steps

Reward standardisation

standardised discipline-specific metadata requirements

discipline specific standardised vocabularies

sustainability

find the smallest possible standard

Think of different standards for different data types

Where/which platform

metadata

Where to share/publish your data -> can that be standardized in a community

Use open formats / Metadata standards / Preregistration / licenses / Open when possible

Minimum metadata requirement / Dublin Core

Documentation (readme file, metadata, code book)

File structure

Policy, standardisation vocabulary, procedure, metadata sheet

Certification of trusted repositories

**Reusability**

Advantages of data reuse:

- saves time
- more efficient data collection
- get more context/inspiration
- unexpected application / serendipity

rich metadata -> more reusability

Obstacles to data reuse

- existing data not (always) FAIR
- findability
- culture
- diversity/complexity = no interoperability

Ways forward / checklist

Reuse

- clear/complete metadata - domain-specific standardisation
- Create synthetic datasets if original data is sensitive
- codebooks (= a legenda of the dataset): standardised, machine-readable
